# The significance of margins in pediatric Non‐Rhabdomyosarcoma soft tissue sarcomas: Consensus on surgical margin definition harmonization from the INternational Soft Tissue SaRcoma ConsorTium (INSTRuCT)

**DOI:** 10.1002/cam4.5671

**Published:** 2023-02-06

**Authors:** Monika Sparber‐Sauer, Andrea Ferrari, Sheri L. Spunt, Christian Vokuhl, Dana Casey, Timothy B. Lautz, William H. Meyer, David O. Walterhouse, Kristian W. Pajtler, Rita Alaggio, Andreas Schmidt, Akmal Safwat, Beate Timmermann, Patrizia Dall'Igna, Sonja Chen, Aaron R. Weiss, Daniel Orbach

**Affiliations:** ^1^ Klinikum der Landeshauptstadt Stuttgart gKAöR, Olgahospital, Stuttgart Cancer Center, Zentrum für Kinder‐, Jugend‐ und Frauenmedizin, Pädiatrie 5 (Pädiatrische Onkologie, Hämatologie, Immunologie) Stuttgart Germany; ^2^ Medizinische Fakultät der Universität Tübingen Tübingen Germany; ^3^ Pediatric Oncology Unit, Fondazione IRCCS Istituto Nazionale Tumori Milan Italy; ^4^ Department of Pediatrics Stanford University School of Medicine Palo Alto California United States; ^5^ Section of Pediatric Pathology, University of Bonn Bonn Germany; ^6^ Department of Radiation Oncology University of North Carolina School of Medicine Chapel Hill North Carolina United States; ^7^ Division of Pediatric Surgery, Department of Surgery Northwestern University Feinberg School of Medicine Chicago Illinois United States; ^8^ Jimmy Everest Section of Pediatric Hematology Oncology, Department of Pediatrics University of Oklahoma Health Sciences Center Oklahoma City Oklahoma United States; ^9^ Division of Pediatric Hematology/Oncology/Stem Cell Transplant, Department of Pediatrics Ann & Robert H. Lurie Children's Hospital of Chicago, Northwestern University Feinberg School of Medicine Chicago Illinois United States; ^10^ Hopp‐Children's Cancer Center, NCT Heidelberg (KiTZ) Heidelberg Germany; ^11^ Division of Pediatric Neurooncology German Cancer Research Center (DKFZ), German Cancer Consortium (DKTK) Heidelberg Germany; ^12^ Department of Pediatric Hematology and Oncology Heidelberg University Hospital Heidelberg Germany; ^13^ Pathology Unit, Department of Laboratories Bambino Gesù Children's Hospital, IRCCS Rome Italy; ^14^ Department of Pediatric Surgery and Pediatric Urology University Children's Hospital, Eberhard Karls University Tuebingen Tuebingen Germany; ^15^ Oncology Department and Danish Center for Particle Therapy Aarhus University Hospital Aarhus Denmark; ^16^ Department of Particle Therapy University Hospital Essen, West German Proton Therapy Centre Essen (WPE), West German Cancer Center (WTZ), German Cancer Consortium (DKTK) Germany; ^17^ Pediatric Surgery, Department of Emergencies and Organ Transplantation University of Bari Bari Italy; ^18^ Department of Pathology and Laboratory Medicine Nationwide Children's Hospital Columbus Ohio United States; ^19^ Department of Pediatrics Maine Medical Center Portland Maine United States; ^20^ SIREDO Oncology Center (Care, Innovation and Research for Children, Adolescents and Young Adults with Cancer), PSL University, Institut Curie Paris France

**Keywords:** margins, pediatric/adolescent oncology, radiotherapy, soft tissue sarcoma, surgery

## Abstract

**Background:**

Margin status following surgery in children, adolescents, and young adults with soft tissue sarcomas is controversial and has been defined differently by various specialties, with definitions changing over time and by cooperative group. The International Soft Tissue Sarcoma Consortium (INSTRuCT) is a collaboration of the Children's Oncology Group (COG) Soft Tissue Sarcoma Committee, European pediatric Soft Tissue sarcoma Study Group (EpSSG), and the European Cooperative Weichteilsarkom Studiengruppe (CWS) devoted to improving patient outcomes by pooling and mining cooperative group clinical trial data.

**Methods:**

The INSTRuCT non‐rhabdomyosarcoma soft tissue sarcoma (NRSTS) working group aimed to develop international harmonized recommendations regarding surgical margin assessment and definitions in children and adolescents with soft tissue tumors.

**Results and Conclusion:**

This review addresses accepted principles and areas of controversy, including the perspectives of surgeons, pathologists, radiation oncologists, and pediatric oncologists, to develop a framework for building common guidelines for future research.

## Graphical abstract

Surgical margin assessment following surgery in children, adolescents, and young adults with soft tissue sarcomas is not standardized, making comparisons of different therapeutic approaches difficult. The International Soft Tissue Sarcoma Consortium (INSTRuCT) addresses accepted principles and areas of controversy, including the perspectives of surgeons, pathologists, radiation oncologists, and pediatric oncologists, to develop a framework for building harmonized margin status guidelines for future protocols.

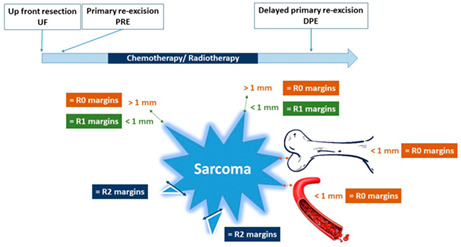

## 1 INTRODUCTION

About 4% of childhood cancers are non‐rhabdomyosarcoma soft tissue sarcoma (NRSTS). Due to its impact on the treatment approach, the definition of “tumor margins” in children, adolescents, and young adults with soft tissue sarcomas (STS) is critical and evaluating and interpreting these margins remains a complex issue with poorly standardized definitions in children and even in adults. The prognostic value of negative tumor margins at diagnosis in pediatric NRSTS has been demonstrated.^1^, ^2^, ^3^, ^4^, ^5^ Nevertheless, different definitions of “margins” and their status are used by various international soft tissue sarcoma working groups, and by different specialties. Definitions for NRSTS margins were originally adapted from rhabdomyosarcoma (RMS) without considering the heterogeneity of the NRSTS subtypes. Pediatric oncologists, surgeons, pathologists, and radiation oncologists may consider “margin” differently according to their own expertize and therapeutic objectives. Similar to RMS, surgeons determine the resectability of the tumor based on initial imaging. Intraoperative findings recorded in the operative note, as well as the handling of the resection specimen by the surgeon, may both influence how the surgical margin is interpreted by the pathologist. Margin status interpretation is also affected by resection specimen processing after removal from the surgical bed and how the specimen is evaluated and reported by the pathologist. Radiation oncologists adjust radiation field size and dose based on the extent of tumor resection, including the width of the margin when negative. Use and sequencing of systemic therapy by the oncologist is influenced by surgical margin status and the consequent need for radiotherapy. Three pediatric oncology cooperative groups, the Children's Oncology Group (COG) Soft Tissue Sarcoma Committee, the European pediatric Soft Tissue sarcoma Study Group (EpSSG), and the Cooperative Weichteilsarkom Studiengruppe (CWS) joined together to establish a combined database within the International Soft Tissue Sarcoma Consortium (INSTRuCT). One of the goals of INSTRuCT is to develop harmonized guidelines on critical issues to be utilized across all cooperative groups for future study development. This review addresses accepted principles and areas of controversy in the assessment and definition of margin status in NRSTS in children and adolescents (age 0–21 years), including perspectives from various cancer subspecialists, in order to harmonize and develop common definitions and guidelines for future research. A goal of seeking consensus across different disciplines on how to assess and define margin status is to simplify and clarify interpretation of outcome data, especially when comparing results from different international groups.

## 2 THE ONCOLOGY PERSPECTIVE: WHAT CLINICAL CLASSIFICATION SYSTEMS ARE USED FOR PEDIATRIC NRSTS?

### 2.1 Clinical classification systems and definitions

Initially, CWS, the EpSSG (the former Malignant Mesenchymal Tumor MMT Committee together with the STSC AIEOP Italian Soft Tissue Sarcoma Committee), and COG used a postsurgical clinical grouping system based on extent of tumor resection adapted from the Intergroup Rhabdomyosarcoma Study Group (IRSG)^4^, ^6^, ^7^ to classify pediatric patients with NRSTS. The IRS system defines Group I—a completely resected localized tumor with negative resection margins; Group IIa—a grossly resected localized tumor with microscopic residual disease; Group IIb—a completely resected localized tumor with resected involved regional lymph nodes; Group IIc—a grossly resected localized tumor with microscopic residual disease together with resected involved regional lymph nodes; Group III—a tumor with gross residual disease after biopsy or after incomplete resection; and Group IV—a tumor with distant metastases.^8^ The IRS grouping system is widely used due to its simplicity^9^ and its prognostic value.^10^, ^11^ Nevertheless, controversies remain regarding definitions of the postoperative extent of disease in NRSTS. Limitations of the IRS grouping system are lack of documentation of margins width and closeness to anatomic boundaries that usually prevent tumor spread. Additionally, this system does not include details on primary re‐excision (PRE) (defined by an immediate wide resection of the primary site before any neoadjuvant therapy) and was not designed to define margins after delayed surgery (defined by a wide local surgery of the primary site after chemotherapy or radiotherapy).

For pretreatment staging, the TNM classification is generally used.^11^ This system includes an estimate of tumor invasiveness, classifying tumors confined to the organ or tissue of origin as T1 and tumors invading adjacent structures as T2, regional nodal status, classifying uninvolved lymph nodes as N0 and involved lymph nodes as N1, and metastasis, M0 if not present and M1 if present.

### 2.2 What is the definition of a negative margins in pediatric NRSTS?

Many adult and pediatric studies categorize margin status after either upfront or delayed surgery according to the American Joint Committee on Cancer (AJCC) residual tumor classification (R‐classification). However, the definitions of postsurgical resection margins within pediatric clinical trials vary among the cooperative groups (Table S1)^12^ and an ongoing challenge is that the extent of negative margins in millimeters is often not available in international databases or from pathology reports. Future use of standardized pathology reporting of margins is needed,^13^ and harmonization of surgical margin definitions is necessary for future studies.^5^ In the COG ARST0332, the 5‐year EFS for young patients with sarcomas and “negative microscopic margins” was ~84% versus 66% with “positive microscopic margins.”^9^ However, this group has previously demonstrated that local control rates were comparable regardless of the size of the negative margins (94% for ≤1 cm margins vs 97% for >1 cm margins) or the anatomic site (96% in upper extremities vs 97% in lower extremities).^14^

Microscopic margin status (positive vs negative) is one of the few reproducible and consistent histologic features which affects long‐term outcome. Negative margins should be the goal, where possible.^5^, ^9^, ^10^, ^15^, ^16^, ^17^ However, North American^9^ and European^10^ trials use different definitions for negative and positive margins. In the COG ARST0332, negative margin (R0) was defined as at least a 5 mm cuff of nonmalignant tissue surrounding the tumor in all directions However, only a minority of pathology reports indicated the width of negative margins and thus the definition was not evaluable. In addition, for sites where tumor abuts fascia/periosteum and is removed in continuity, resection was considered complete (R0). Since the width of the negative margin was often not specific, though, it was difficult to ensure compliance with protocol guidelines for a 5 mm margin to be considered R0. In contrast, in the EpSSG NRSTS 2005 protocol and the CWS guidance a definition of negative margin in which resection is performed through normal tissue beyond the tumor and its pseudocapsule (wide resection) without an assigned number of millimeters from the margin (R0) (Table S1). Traditionally, in the EpSSG NRSTS protocols, adequate margins have been defined as ≥1 cm of healthy tissue around the tumor in all directions for muscle, or >1 mm of healthy tissue around the tumor when the tissue is periosteum, vessel sheath, epineurium, or muscular fascia. Past COG protocols considered margins <0.5 cm without any fascia to be “marginal” and those ≥0.5 cm as “wide resection.” More recent CWS and COG protocols do not specify a specific margin width.

The *“R‐classification”* allows margins closer than 1 mm to be regarded as R0, whereas the *“UICC‐classification”* (*Union internationale contre le cancer*) considers margins closer than 1 mm to be positive (R1). Kainhofer et al. assessed the impact of margin status according to these systems and found that the margin status represented an independent prognostic factor for local recurrence (LR) in patients with STS following curative surgery.^18^ Local control rates were superior after a minimal resection margin of 1 mm (R0 by UICC‐classification) compared to <1 mm (R0 by the R‐classification).

Even if the definition of” negative margin” is variable, the prognostic value of this risk factor remains significant in most studies, with better outcomes for patients after “complete primary resection” than in cases of “incomplete surgery.”.^3^, ^9^, ^10^, ^19^

Another point of debate concerning the IRS grouping system is the interpretation of group III status, that is, macroscopic tumor residual after biopsy or surgery. Once again, the philosophy of the cooperative groups differs. COG protocols recommend delayed resection for >5 cm high‐grade tumors, including localized tumors amenable to complete resection, as they will receive RT regardless of extent of upfront resection except in the setting of amputation. In this case, administering preoperative RT may have the advantage of limiting the dose and size of the field required.^20^ For >5 cm tumors and certain NRSTS histotypes known to be chemosensitive, EpSSG and CWS prefer an upfront biopsy with a delay of local therapy after chemo‐reduction. The use of RT and the size of the RT fields depend on the tumor resectability and the timing of RT (pre‐, postoperative, or definitive) is often decided in a multidisciplinary team discussion. Consequently, the management of IRS group III tumors is heterogeneous: some could be considered “unresectable” and others “unresected” (but resectable after neoadjuvant therapy). Indeed, “unresectable” tumor depends on the surgeon's assessment, which may vary depending on experience and skill. In the ARST0332 study, COG defined tumors eligible for upfront chemotherapy‐RT to include both unresectable tumors according to the surgeon and >5 cm high‐grade tumors that were anticipated to have inadequate microscopic margins after upfront surgery. However, many patients in the latter group underwent upfront surgery prior to study entry, further complicating comparisons of outcome.

For the pediatric oncologist, another question to address is whether the definition of adequate margins should vary according to histologic subtype. The extent of negative margin may differ according to the behavior of the tumor: low‐ or intermediate‐grade tumors might require more limited surgical margins than large, high‐grade tumors. The possibility of complete resection at diagnosis varies among histotypes (Table 1)^9^, ^10^, ^21^, ^22^, ^23^, ^24^, ^25^, ^26^, ^27^, ^28^, ^29^, ^30^, ^31^, ^32^ and by tumor grade, but may also be affected by the size of the patient. For instance, infantile fibrosarcoma (IFS) may have a low likelihood of complete resection because it often presents as a relatively large tumor in babies (median age: 1.9 months), rather than being a particularly locally invasive tumor such as desmoid type fibromatosis. In an analysis of 96 young patients with low‐ and intermediate‐grade soft tissue tumors, initial margin status did not impact outcome (IRS group I vs II vs III/IV; *p* = 0.194) with a 5‐year locoregional relapse‐free survival (LRRFS) of 92.6% (95% Confidence Interval, 87.1–98.5) for the whole cohort.^33^ Conversely, obtaining negative margins in high‐grade sarcomas, such as epithelioid sarcomas, is important to minimize the risk of local recurrence.^34^ In conclusion, defining the adequacy of surgical margins must account for both the biologic features of the specific tumor type and the patient‐specific clinical context. Moreover, the COG ARST0531 rhabdomyosarcoma protocol highlights the fact that systemic therapy may also contribute to local control and that this can potentially have an effect on EFS/OS.^8^, ^35^, ^36^

**TABLE 1:** Literature on margins in pediatric patients with NRSTS.

Disease entity	References	Median age (ranges)	Patient numbers	Complete tumor resection at diagnosis—IRS I (%)
All type of NRSTS (only localized tumors)	10	12.6 years (0–21)	569	252 (44%)
	9	13.6 years (0.1–29.8)	529	252 (48%)
High frequency of complete resection (> 70%)				
Protuberans dermatofibrosarcoma	49	8 years (0.64–17.7)	40	28 (70%)
	21	6.9 years (0.4–17.5)	46	35 (76%)
Intermediate frequency of complete resection (30%–70%)				
Infantile fibrosarcoma	22	0.14 years (0.0–1.79 years)	66	17 (26%)
	23	1.4 months (0.03–18.73 months)	50	27 (54%)
	24	2.6 months (0–24 months)	56	37 (66%)
Inflammatory myofibroblastic tumors	56	9.5 years (0.2–24)	60	31 (52%)
Epithelioid sarcoma	22	14 years (0.7–26.9)	67	5 (52%)
	25	13.1 years (2.7–24.8)	63	32 (51%)
Alveolar soft part sarcoma	26	13 years (2–21)	51	18 (35%)
	27	14 years (3–20)	61	38 (62%)
Low frequency of complete resection (< 30%)				
Synovial sarcoma (only localized tumors)	28	13.7 years (not specified‐21)	138	41 (30%)
MPNST (only localized tumors)	32	13.7 years (0.02–21.3)	51	13 (25%)
Desmoid fibromatosis type	29	11.4 years (0.1–24)	173	24 (14%)
	30	9.48 years (0.02–18.05)	90	Not specified
Renal and extrarenal rhabdoid tumor	31	Not specified	18	0 (0%)

While this does not completely address the question of how many millimeters is required to ensure a very low likelihood of local recurrence, a 5 mm margin seems a reasonable goal when feasible, but there is little evidence that such a large margin is necessary or attainable in all anatomic locations or in small children.

### 2.3 What is the reported relationship of margin width and risk of local recurrence in pediatric NRSTS?

The question that arises is whether there is a difference in outcome for patients with R1 specimens wherein the tumor cells are at the margin (or tumor cells touching the inked margin) versus those in which there were tumor cells 1 mm, 2 mm, 3 mm, or 4 mm away from the inked margin. There is no published data at this time that can provide an answer to this question, however based on prior evidence,^19^, ^37^ the possible conclusion is that a negative margin (no tumor cells at ink) is necessary, and the larger the negative margin, the lower the risk of LR.^9^, ^10^, ^14^, ^38^, ^39^ In adults, no clear data exist to define a correlation between a “surgical safe margin depth” and “pathology safe margin width” despite many attempts and retrospective analyses.^38^, ^40^, ^41^, ^42^, ^43^ The prognostic value of margins in millimeters has been evaluated in few studies.^44^ In adults with primary extremity STS, LR rates were only 8% when margins were >2 mm, and increased when margins were <1 mm. Classifying margins as R1 or R0 seems more discriminating than measurements of negative margins in millimeters, however, the role of neoadjuvant treatment and tumor biology also need to be considered.^45^, ^46^, ^47^ Moreover, the quality of margins has been improved over the past 20 years by the more frequent use of adjuvant and neoadjuvant treatments^48^, ^49^, ^50^, ^51^ and the wider practice of reconstructive surgeries (42.2% with flap reconstruction in a recent experience).^52^

## 3 THE SURGICAL PERSPECTIVE

### 3.1 What surgical principles should guide decisions about upfront resection, primary re‐excision, and delayed primary excision following neoadjuvant therapy?

In general, the goal of tumor surgery is to obtain an R0 resection while preserving form and function.^53^ For sarcomas, surgery that leaves gross residual tumor (R2) is discouraged due to poor outcomes for these patients, and should generally be followed by re‐excision when feasible. The ability to perform en bloc resection with wide margins varies based on location and patient age and size. In some surgical sites, such as the head and neck, it is challenging to obtain completely negative microscopic margins.^54^

Surgical excision should include an en bloc resection of the biopsy site. The specimen should be clearly marked and oriented for pathologic assessment. If there is any intraoperative concern about proximity to a specific margin, an additional margin specimen should be resected and sent for frozen section evaluation. For tumors abutting bone, excision should include the adjacent periosteum. Likewise, fascia should be excised when abutted by tumor, but the dissection does not need to extend into healthy muscle in adjacent compartments. If margins along periosteum, epineurium or major blood vessels are microscopically positive, then additional radiation may be considered. Titanium clips, which do not to interfere with CT or MRI scans, should be placed to mark the periphery of the resection field, and especially to mark areas of close margins in order to guide adjuvant RT. Clips should be placed in the most cranial, caudal, medial, and lateral margins of the tumor bed, as well as in any areas of residual tumor.

Adequate preoperative imaging is essential for surgical planning. Magnetic resonance imaging (MRI) is the modality of choice for most soft tissue lesion sites and delineates tumor relationships to fascial planes and neurovascular structures. For selected anatomic regions, CT scan and other imaging modalities may be necessary. In cases where the tumor is adherent to major neurovascular structures, careful preoperative discussion with the family, oncology, and radiation oncology teams, taking into account the chemo‐ and radiosensitivity of the specific NRSTS histologic subtype, the anatomic location, and the anticipated risk of postoperative wound healing complications following preoperative radiotherapy can guide decisions about whether surgery should occur upfront or after neoadjuvant therapy.^55^

Primary re‐excision is intended to achieve a wide resection while still preserving function, and should include the scar and the superficial and deep tissues left behind during the previous surgery.^56^ PRE should be considered for patients with NRSTS as studies in children and adults with STS have described the usefulness of PRE in patients with residual disease.^57^ The importance of PRE was better documented in NRSTS where PRE improved resection status in 69%.^58^ Other studies on the importance of PRE in adults with STS showed that positive tumor margins compared with negative margins strongly predicted LR risk (*p* < 0.01) with a hazard ratio of 3.76 (95% confidence interval [CI]: 2.20–6.40) in these tumors, which are often poorly sensitive to chemotherapy. In the pediatric series from Cecchetto et al., the results of PRE seemed to be linked to the size of the tumors, and local recurrences were more frequent for tumors larger than 5 cm and in some specific sites.^16^

All patients who do not undergo upfront resection or PRE should be assessed for possible delayed excision after neoadjuvant chemotherapy and/or radiation. As described above, COG protocols recommend DPE for high‐grade tumors >5 cm, since these patients will receive RT regardless of resection status. Likewise, any patient with any initially unresectable tumor should be reassessed after induction therapy to determine feasibility of DPE.

### 3.2 Are there intraoperative visualization techniques that can be used to optimize chances of negative surgical margins in NRSTS?

Intraoperative ultrasound (US) used by experts might be a useful tool for achieving negative margins for tumors that are non‐palpable, ill‐defined, or beneath the fascia. In one study of 19 patients with malignant sarcomas, intraoperative US was helpful for visualizing tumor borders, and facilitated an R0 resection in 95% of cases.^59^ Intraoperative fluorescence‐guided surgery (FGS) is a promising new modality which may help reduce rates of inadvertent positive margins. In a study of 39 adult patients with sarcoma who underwent near‐infrared (NIR) FGS with indocyanine green (ICG), compared to 76 undergoing standard surgical technique, patients receiving ICG had a lower unexpected positive margin rate (5.1% vs 25.0%, *p* = 0.01).^60^ In this study, the tumor was noted to be fluorescent in 37 of 39 cases, and the surgeon deemed the fluorescence to have helped guide the surgery in 11 cases. Novel fluorophores may offer even more precise and reliable option for utilizing NIR‐FGS to delineate sarcoma margins.

## 4 THE PATHOLOGY PERSPECTIVE

### 4.1 How are margins assessed by the pathologist in NRSTS?

Pathologic assessment of NRSTS is crucial to ascertain tumor size, margin status, completeness of resection and, in cases where neoadjuvant therapy is utilized, treatment effect. Primary resection provides the most accurate assessment of the tumor in relation to its margins. An important point concerning margins is the difference between assessment of intraoperative margins on fresh tissue and assessment of margins on formalin‐fixed tissue: the ratio between the two is 2:1.^61^ Therefore, achieving an intraoperative margin of at least 1 cm is necessary for a 5 mm tumor‐free margin in the formalin‐fixed specimen. Most American‐based pathology programs ink the specimen resection margins and ask surgeons to provide details on specimen orientation. It is advised to submit margins perpendicular to the tumor, along with a minimum of 1 section per cm of the tumor.^62^ Sampling should also include areas that appear “different” such as firmer in appearance, in the case of possible liposarcoma; or solid areas in the case of a blastemal tumor. Sampling different grossly appearing areas is often the key to identification of a higher grade component or revealing the extent of treatment effect in a delayed resection specimen.^63^ A major challenge is to ascertain the negative margins in multinodular lesions (i.e., low‐grade fibromyxoid sarcoma) or infiltrative lesions. Additionally, when neoadjuvant therapy is used, native tumor necrosis or fibrosis versus tumor necrosis or fibrosis due to treatment effect can be challenging to distinguish histologically.

### 4.2 What pathology information should standardly be reported with every NRSTS resection?

In the United States, the College of American Pathologists (CAP) provides standard reporting templates to guide pathologists in providing useful diagnostic and prognostic information from NRSTS resection specimens. In one study, a review of such reports in childhood NRSTS documented critical factors influencing treatment such as margin status in 92% of reports, whereas pathologic grade was documented in only 40% of evaluable reports.^13^ In addition, the International Collaboration on Cancer Reporting (ICCR) established standardized tumor reporting templates including soft tissue sarcoma which should alternatively be used to provide uniform pathology reporting regardless of tumor grade. With every NRSTS the depth/width of the pathology margins must be documented precisely, and margins that fall across areas of tumor necrosis or margins adjacent to anatomic boundaries that naturally limit tumor spread such as fascia must be clearly identified. Moreover, information on the margin status for primary delayed resection that were achieved post‐chemo, post‐RT, or both must be detailed (Table 2).The North American and European trials in pediatric patients with NRSTS established the diagnosis of these soft tissue tumors initially using the 2002 World Health Organization (WHO) criteria, while utilizing two different grading systems, the Pediatric Oncology Group (POG) and the *Fédération Nationale des Centres de Lutte Contre le Cancer* (FNCLCC), respectively.^9^, ^10^, ^47^, ^64^, ^65^ It should also be noted that certain histologies which were included in COG ARST0332 such as undifferentiated sarcomas, and hepatic undifferentiated embryonal sarcomas, were excluded from the pediatric NRSTS EpSSG trial and the Soft Tissue Sarcoma registry SoTiSaR (Table S2). Evaluating the relevance of margin status must therefore be considered in the context of the differing grading guidelines used to define tumor biologic behavior as well as treatment approach alterations that result from these differences.^9^, ^10^, ^66^

**TABLE 2:** INSTRuCT proposal for common definitions of pediatric NRSTS surgical margins.

For clinical trial and research purposes, surgical margin status should be defined with the input of the surgeon(s) involved in each operation as well as the pathologist who examined the gross specimen and the microscopic margins.
Surgical margin status uses the R system, which separates tumors into three broad categories by the pathologist:
R0: At least 1 mm of nonmalignant tissue between the tumor and the surgical margin following en bloc resection without tumor spillage. Also included are tumors where malignant cells are within 1 mm of an uninterrupted anatomic boundary such as fascia and any other anatomic barriers (e.g., periosteum, vessel sheath, and epineurium), in which the AB designation should be added: R0 (AB). R1: Tumor cells within 1 mm of the surgical margin following grossly complete tumor resection. Tumors with any viable cells within 1 mm of the surgical margin should carry the M designation: R1 (M), whereas tumors with only necrotic tumor cells (not fibrosis) within 1 mm of the surgical margin should carry the N designation: R1 (N). R2: Gross residual tumor.
Additional clinical designations indicated by the clinical research associate should include:
R2 (A): Subtotal tumor resection; residual tumor potentially amenable to gross total resection. R2 (B): Subtotal tumor resection; gross total tumor resection not possible without mutilation. R2 (C): Resection not performed; gross total tumor resection not possible without mutilation. R2 (X): Surgical procedure unknown.
The timing of the surgical procedure is added in square brackets after the R status and additional designation(s):
[UF]: Upfront [PRE]: Primary Re‐excision: Resection immediately following an initially incomplete primary resection and before chemotherapy or radiotherapy. [DPE]: Delayed Primary Re‐excision: Resection after neoadjuvant chemotherapy and/or radiotherapy
Examples Unresectable tumor at initial diagnosis, treated with chemotherapy and then completely resected with negative margins—at least one of which included tumor touching an intact fascial boundary: R2(B)[UF]/R0(AB)[DPE]. 2) Subtotal resection with residual tumor potentially amenable to gross total resection, followed by a second surgery that removed all gross tumor but viable tumor evident <1 mm from the surgical margin: R2(A)[UF]/R1(M)[PRE].

## 5 RADIOTHERAPY CONSIDERATIONS

RT can be utilized either in the neoadjuvant or adjuvant setting.^5^, ^20^ The widespread adaptation of less aggressive surgery has led to the increased use of planned pre‐ or postoperative radiotherapy. There are also data suggesting that omission of RT in certain settings in pediatric patients may be safe, including <5 cm high‐grade tumors with R0 margins^9^, ^67^ and low‐grade tumors with R1 margins.^9^, ^68^ This highlights the importance of careful assessment of tumor factors and margins in determining whether RT is needed.

### 5.1 How do factors as timing of surgery impact radiotherapy decisions for patients with NRSTS?

Regarding the timing of RT, 45 Gy RT delivered preoperatively in combination with chemotherapy results in a high likelihood of R0 margins at delayed surgery, thereby limiting both dose and volume required.^69^, ^70^ Thus, sequencing RT before surgery is the preferred approach through the COG^20^ in those anticipated to require RT and may be helpful to achieve negative margins with a lower dose and smaller field size, which are both important considerations in pediatric patients. In Europe, the randomized study FaR‐RMS (NCT04625907) addresses the question on pre‐ versus postoperative RT in localized disease of rhabdomyosarcoma. Even though the data in pediatric patients are limited, available evidence suggests that RT has similar benefits in pediatric and adult patients for local tumor control, especially in those with positive margins. Toxicity of RT is important to consider, especially in children due to incomplete growth and development and long duration of life during which the risk of secondary malignancy continues to increase with time.

### 5.2 What definitions are used for RT planning in pediatric NRSTS?

For RT planning in NRSTS in both the neoadjuvant and adjuvant setting, clear definitions and delineation of the gross tumor volume (GTV), clinical target volume (CTV), and planning target volume (PTV) are critical (Table 3). In general, for pediatric NRSTS, the GTV consists of the initial tumor volume at diagnosis (visible via imaging such as with MRI and clinical exam). If induction chemotherapy or surgical resection has been performed prior to RT planning, the GTV should be modified to account for the return of normal tissues to their original position, but must include all infiltrative disease at initial diagnosis. In addition, in the postoperative setting specifically, the GTV should include any sites of microscopic or residual gross disease as defined by the operative report, pathology report, and postoperative imaging, specifically taking care to include any sites of close or positive margins and considering the biopsy tract/drainage/surgery scars. Systematic inclusion of scars in the radiation field may not be justified.^71^ Standardizing RT practice across all soft tissue sarcoma groups on all aspects of RT could improve the ability to compare local control outcomes. When one variable is different between the groups (e.g., dose to the GTV or size of the CTV relative to the GTV), it limits the ability to compare outcomes based on surgical margin status across groups.^72^, ^73^, ^74^, ^75^, ^76^

**TABLE 3:** Definition of radiotherapy volumes and margins for pediatric soft tissue sarcomas in EpSSG RMS‐2005, COG ARST 0332, and CWS Guidance.

	COG ARST0332	EpSSG: NRSTS 05/RMS‐2005	CWS: CWS‐Guidance (2012)
Target volume definition for primary tumor—Gross tumor volume (GTV)	The volume occupied at diagnosis by visible or palpable disease.	Chosen according to the initial tumor volume on pre‐therapeutic T1 MRI image with contrast. Exceptions: intrathoracic or pelvic tumor bulk.	Idem EpSSG.
Clinical target volume (CTV)	Includes the GTV and sites with potential occult tumor involvement including lymph nodes adjacent to the GTV that may be clinically involved.	Defined as the GTV + 1 cm (Exception limbs: 2 cm in longitudinal directions). Additionally, scars of the biopsy, of the initial surgery, of the second look surgery and of drain sites have to be included in the CTV. All tissues that were potentially tumor contaminated during surgery need to be included in the CTV.	Idem EpSSG
Planning target volume (PTV)	The CTV surrounded by a geometric margin to account for variability in setup, breathing, or motion during treatment.	Defined as the CTV + 1 cm (Exception chest wall: 2 cm) In patients receiving a boost after 50.4 Gy, the PTV of the boost is the residual tumor at the start of radiotherapy plus a margin of 1–2 cm.	Defined as the CTV + 5–10 mm. *Exceptions* : Chest wall: 2 cm. In patients receiving 50.4 Gy, the CTV and hence the PTV is reduced to the GTV (initial tumor) plus 5–10 mm after 41.4 Gy. In patients with *orbital tumor*, the reduction of the target volume is performed after 36 Gy. In patients receiving a boost after 50.4 Gy, the PTV for the boost is the GTV of the residual tumor at the start of radiotherapy plus a margin of 5–10 mm.

## 6 CONCLUSIONS

Recommendations for defining the adequacy of surgical margins in pediatric NRSTS patients is complex and depends on tumor biologic behavior, the surgical approach used, the way tumor was handled intraoperatively and postoperatively, the pathologic approach to margin interpretation, as well as whether neoadjuvant therapy was administered.^38^, ^48^ Existing systems for assessing surgical margins lack the consistency and detail required to precisely study questions of margin adequacy. To answer unresolved questions and address major deficiencies in the existing systems for margin classification, the authors propose a new margin classification system. This new system includes information on PRE and the difference between “unresected tumor” which potentially can be resected by re‐excision and “unresectable tumor.” Furthermore, the depth/width of the margins must be documented precisely in the pathology report, and should include areas of tumor necrosis at the margin as well as margins adjacent to specific anatomic boundaries. Moreover, information on the margin status for primary delayed resection that were achieved post‐chemotherapy, post‐RT, or both must be detailed. The tumor grade and innate tumor biology highlight the heterogeneity of NRSTS and these data should be collected by registries and analyzed in future trials to further inform prognostic value and treatment methods. Although the authors acknowledge that some important data are lacking to optimally define “margins” in pediatric NRSTS, we have proposed consensus definitions to serve as a tool for future prospective studies (Table 2).

## AUTHOR CONTRIBUTIONS

**Monika Sparber‐Sauer:** Conceptualization (lead); data curation (equal); formal analysis (lead); methodology (equal); project administration (equal); supervision (equal); validation (equal); visualization (equal); writing – original draft (equal); writing – review and editing (equal). **Andrea Ferrari:** Conceptualization (equal); formal analysis (equal); methodology (equal); project administration (equal); supervision (equal); validation (equal); writing – review and editing (equal). **Sheri L Spunt:** Conceptualization (equal); formal analysis (equal); methodology (equal); validation (equal); visualization (equal); writing – original draft (equal); writing – review and editing (equal). **Christian Vokuhl:** Formal analysis (equal); methodology (equal); supervision (equal); validation (equal); visualization (equal); writing – review and editing (equal). **Dana Lynne Casey:** Conceptualization (equal); methodology (equal); supervision (equal); validation (equal); writing – original draft (equal); writing – review and editing (equal). **Timothy B Lautz:** Methodology (equal); supervision (equal); validation (equal); visualization (equal); writing – original draft (equal); writing – review and editing (equal). **William H Meyer:** Formal analysis (equal); methodology (equal); supervision (equal); validation (equal); writing – review and editing (equal). **David O. Walterhouse:** Formal analysis (equal); methodology (equal); supervision (equal); validation (equal); visualization (equal); writing – original draft (equal); writing – review and editing (equal). **Christian W Pajtler:** Methodology (equal); validation (equal); visualization (equal); writing – original draft (equal); writing – review and editing (equal). **Rita Alaggio:** Conceptualization (equal); methodology (equal); validation (equal); visualization (equal); writing – original draft (equal); writing – review and editing (equal). **Andreas Schmidt:** Methodology (equal); supervision (equal); validation (equal); visualization (equal); writing – review and editing (equal). **Akmal Safwat:** Methodology (equal); supervision (equal); validation (equal); writing – original draft (equal); writing – review and editing (equal). **Beate Timmermann:** Conceptualization (equal); methodology (equal); validation (equal); visualization (equal); writing – original draft (equal); writing – review and editing (equal). **Patrizia Dall'Igna:** Methodology (equal); validation (equal); visualization (equal); writing – original draft (equal); writing – review and editing (equal). **Sonja Chen:** Conceptualization (equal); methodology (equal); validation (equal); visualization (equal); writing – original draft (equal); writing – review and editing (equal). **Aaron Weiss:** Conceptualization (equal); formal analysis (equal); methodology (equal); supervision (equal); validation (equal); visualization (equal); writing – original draft (equal); writing – review and editing (equal). **Daniel Orbach:** Conceptualization (equal); formal analysis (equal); investigation (equal); methodology (equal); project administration (equal); supervision (equal); validation (equal); visualization (equal); writing – original draft (equal); writing – review and editing (equal).

## FUNDING INFORMATION

The International Soft Tissue Sarcoma Consortium and the Pediatric Cancer Data Commons are supported in part by the Cancer Research Foundation, Children's Research Foundation, Comer Development Board, KickCancer, King Baudouin Foundation, Rally Foundation for Childhood Cancer Research, Seattle Children's Foundation from Kat's Crew Guild through the Sarcoma Research Fund, St Baldrick's Foundation, and The Andrew McDonough B+ Foundation.

## CONFLICT OF INTEREST STATEMENT

The authors declare that there are no conflicts of interest concerning this article.

## Supporting information


Table S1.

Table S2.

## Data Availability

Availability of data: Main data analysed in this manuscript are available in published articles (and its supplementary files).
