# Supplementary material for: The significance of margins in pediatric Non‐Rhabdomyosarcoma soft tissue sarcomas: Consensus on surgical margin definition harmonization from the INternational Soft Tissue SaRcoma ConsorTium (INSTRuCT)
Source: Cancer Med. 2023 Feb 6;12(10):11719–30. doi: 10.1002/cam4.5671 (PMC10242312; doi:10.1002/cam4.5671)
Supplement: Supplementary file 1 — Table S1. [file CAM4-12-11719-s001.docx]

|  | **COG ARST0332** | **EpSSG: NRSTS 05** | **CWS: CWS-Guidance (2012)** |
| --- | --- | --- | --- |
| R0 resection (microscopically complete resection = radical resection) | Negative: a cuff of non-malignant tissue measuring at least 5 mm in all directions surrounds the tumor in the operative specimen (Wide resection). When the tumor abuts fascia or periosteum and the fascia or periosteum is removed in continuity with the tumor specimen, this margin will also be considered negative.  A radical resection was defined as removal of the entire anatomic compartment containing the tumor. | - **Wide resection**: It is an en-bloc resection through normal tissue, beyond the reactive zone, with the removal of the tumour with its pseudocapsule and a margin of normal tissue; a resection could be defined as “wide” when the tumour is covered at every point by healthy tissue (muscle, subcutaneous tissue, thick fascia or intermuscular septum) according to the growth pattern of the tumour. When the tumour involves more than one anatomical compartment, the wide resection may include adjacent muscle compartment, bone, blood vessels or nerves (…)  − **Compartmental surgery**: When the tumour is removed en-bloc with the entire muscular or anatomical compartment and is covered by intact deep fascia. This surgery is feasible when tumour is entirely anatomically confined. | R0 resection is defined as complete (or radical) resection of the tumour with adequate safety distance showing histologically tumour free margins without evidence of microscopic residues.  **Wide resection:** Wide resection means en-bloc resection of the tumour with its pseudocapsule, the reactive inflammatory zone and with a margin of normal tissue. The resection can be defined as “wide” when the tumour is covered at every point by sufficient healthy tissue (muscle, subcutaneous tissue, thick fascia, periosteum or intermuscular septum) according to the growth pattern, which can be delimitable, invasive in continuity or invasive in discontinuity (skip lesions). If the tumour involves more than one anatomical compartment the “wide resection” may include several adjacent muscle compartments, bone, blood vessels and/or nerves.  **Compartmental resection***:* This term mainly applies to surgery of the extremities mostly in soft tissue sarcomas limited to one anatomical muscular compartment, which can be completely resected from origin to insertion including surrounding fascial layers. |
| R1 resection (microscopically incomplete = marginal resection) | Positive: the cuff of non-malignant tissue surrounding the grossly completely excised tumor in the operative specimen is < 5 mm in thickness in one or more plane. | When the tumour surface emerges macroscopically at the resection surface (e.g. surgical plane through the reactive zone or pseudo-capsule), or when microscopic tumour extension is present at the margin of resection, but without evidence of macroscopic disease residue.  Surgery is defined contaminated when accidental rupture of the tumour pseudocapsule with spillage of material into the operating field occurs, and when the pseudocapsule has simply emerged at the margin of resection. | R1 resection is defined by microscopic extension of the tumour to the resection margin (or surgical plane through the reactive zone or pseudo-capsule) without evidence of macroscopic residues. Contamination may also be considered as R1-resection.  **Contamination:** Contamination means accidental rupture of the pseudocapsule of the tumour with spillage of tumour material into the operating field. Microscopic tumour contamination is then unavoidable. This spillage of tumour material must be controlled by resection of the whole contaminated area. Microscopic tumour contamination has also to be suspected if the pseudocapsule has emerged to the margin of resection. In this case, the margins have to be extended and the contaminated area has to be resected. It is up to the judgement of the surgeon to decide if the additional excision of the contaminated area was or was not sufficient. In case of doubt, the resection after tumour contamination has to be classified as R1 resection. |
| R2 resection (macroscopically incomplete resection = intralesional resection) | Gross residual tumor after maximal resection | When macroscopic tumour residue is left in situ. | Defined by macroscopic extension of the tumour to the resection margin with evidence of macroscopic residues. |
| Specific considerations |  | A layer of healthy tissue between tumour and resection margins should exist. This layer of healthy tissue is defined as a “safety distance”, and depends on the type of the tissue. Adequate margins have been defined as: >1 cm of healthy tissue around the tumour in all directions (when the tissue is a muscle), >1 mm of healthy tissue around the tumour when the tissue is periostium, vessel sheath, epineurium, muscular fascia. |  |
| Primary resection | Gross total resection required *except for:*   - Non-metastatic high-grade tumor > 5 cm and gross/microscopic residual tumor anticipated after resection - Low- or high-grade tumor that cannot be grossly excised without unacceptable morbidity - High-grade tumor with metastases | Primary resection is indicated:  1. If there is no clear clinical evidence of lymph node or metastatic disease  2. If the tumour can be excised with adequate margins and without danger or mutilation. | **Aim:** To achieve complete resection (R0) in patients with macroscopic or microscopic - certain or doubtful – tumour residue after primary biopsy or primary inadequate operation, before any other therapy, if this can be done without mutilation or functional impairment. |
| Secondary (delayed) resection | Delayed resection planned after 12 weeks of concomitant chemotherapy/radiotherapy for all patients not qualifying for primary resection. | If a primary marginal excision or excisional biopsy (not recommended) has already been done, or where histological evaluation is inadequate, then primary re-excision should be considered. In case of adequate margins (or no tumour) on specimen from primary re-excision, patient could be classified as IRS Group I only if the description of first surgery allows to be confident that no tumour spill and contamination has occurred. | **Aim:** To achieve local tumour control by resection (R0, R1) of any residual mass after neoadjuvant chemotherapy in combination with preoperative or postoperative radiotherapy. |

**Supplementary Table 1**. Definitions of surgical/pathological margins in COG ARST 0332, EpSSG NRSTS-05, protocols and CWS Guidance.
