# Supplementary material for: The significance of margins in pediatric Non‐Rhabdomyosarcoma soft tissue sarcomas: Consensus on surgical margin definition harmonization from the INternational Soft Tissue SaRcoma ConsorTium (INSTRuCT)
Source: Cancer Med. 2023 Feb 6;12(10):11719–30. doi: 10.1002/cam4.5671 (PMC10242312; doi:10.1002/cam4.5671)
Supplement: Supplementary file 2 — Table S2. [file CAM4-12-11719-s002.docx]

|  | **EpSSG NRSTS 05**[**^10^**](#_ENREF_10) | **COG ARST0332**[**^9^**](#_ENREF_9) | **SoTiSaR (2009-2021)^#^** |
| --- | --- | --- | --- |
| **Tumor types evaluated** |  |  |  |
| Synovial sarcoma | 206 | 138 | 147 |
| Adult type NRSTS | 363 | 226 | 271 |
| MPNST | 74 | 58 | 75 |
| Liposarcoma | 35 | 25 | 15 |
| Dermatofibrosarcoma protuberans | 64 | 21 | 50 |
| Low grade fibromyxoid sarcoma | 18 | 18 | 37 |
| Leiomyosarcoma | 22 | 10 | 4 |
| Angiosarcoma | 13 | 6 | 13 |
| Clear cell sarcoma of soft tissue | 17 | 7 | 17 |
| Epithelioid sarcoma | 34 | 28 | 23 |
| Alveolar soft part sarcoma | 19 | 24 | 14 |
| Fibrosarcoma, adult type | 8 | 1 | 7 |
| Undifferentiated pleomorphic sarcoma (Malignant fibrous histiocytoma) | 7 | 16 | 13 |
| Unclassified soft tissue sarcoma | 52 | 44 | 3 |
| Undifferentiated sarcoma | 36* | 47 | 55 |
| Undifferentiated Embryonal Sarcoma, liver | 40* | 39 | 23 |
| Other specific entity | 595* | 47 | 897*** |
| **Number of patients** |  |  |  |
| Enrolled in study/ registry | 1291 | 588 | 1315 |
| At study entry/Eligible | 569 | 529** | 418 |
| Completed treatment | 569 | 433 | 418 |
| **POG Grade** |  |  |  |
| 1 | - | 60 | 12 |
| 2 | - | 86 | 40 |
| 3 | - | 383 | 35 |
| **FLNCC Grade** |  |  |  |
| 1 | 148 | 70 | 16 |
| 2 | 115 | 222 | 24 |
| 3 | 165 | 236 | 13 |
| Missing/indeterminate | 141* | 1 | 278 |
| **Tumor size** |  |  |  |
| ≤5 cm | 301 | 195 | 205 |
| >5cm | 258 | 334  - | 165 |
| Missing/indeterminate | 10* |  | 48 |
| **Extent of Primary Tumor Resection** |  |  |  |
| IRS I/R0 | 252 | 252 | 157 |
| IRS II/R1 | 108 | 81 | 99 |
| IRS III/R2 or unresected | 209 | 196 | 162 |
| **TOTAL** | **569** | **529**** | **418** |

*not included in study analysis; **550 eligible, 529 eligible and evaluable; ***including benign tumors; ^#^unpublished data

**Supplementary Table 2.** Comparison of European (EpSSG NRSTS 05, SoTiSaR) and COG (ARST 0332) pediatric NRSTS trials characteristics.
